# Challenges in contactless temperature determination of supercooled aqueous droplets

**DOI:** 10.1073/pnas.2511453122

**Published:** 2025-09-26

**Authors:** Tillmann Buttersack, Sigurd Bauerecker, Thomas Leisner

**Affiliations:** ^a^Fritz Haber Institute of the Max-Planck Society, Berlin 14195, Germany; ^b^Institute of Physical and Theoretical Chemistry, Technical University of Braunschweig, Braunschweig 38106, Germany; ^c^Institute of Meteorology and Climate Research, Karlsruhe Institute of Technology, Eggenstein-Leopoldshafen 76344, Germany

Biswas et al. reported on a promising experimental setup to monitor the freezing of levitated and supercooled aqueous droplets with volumes between 1 and 12 µL with a multispectroscopic approach (visible, infrared, Raman) (1), which combines previously applied approaches (2–5). This phase transition, even though not the first stage of cloud formation, is ubiquitous in the atmosphere and, therefore, subject to research since decades. Herewith, we would like to point out an apparent misinterpretation of the presented data and also highlight potentially overlooked potential of the experimental setup.

Accurate temperature measurements are challenging, in particular, when done contactless and when a small metastable system is in the focus. However, the presented setup allows for three different ways to evaluate the temperature contactless. While the estimation via Newton’s cooling law requires the heat transfer coefficient for the particular system and knowledge about temperature of the surrounding [furthermore evaporative cooling (low relative humidity) must be considered in this case] (4, 6), the used infrared imaging can, e.g., suffer from background radiation and the geometry of the object (7).

Surprisingly, the authors reported (figure 2D) that larger droplets could be supercooled more than smaller ones. However, this observation was not explained or compared to other studies, which indicate homogeneous nucleation is volume-proportional and surface nucleation may only be relevant for tiny droplets (8). Heterogonous nucleation depends mainly on the time the droplets spend in the chamber and larger droplets are always warmer after a given time. Potentially, the temperature measurement via IR camera is distorted more for smaller droplets than for larger ones, since in figure 1C strong (apparent) temperature gradients of about 20 K/mm are visible (which value was used?). This distortion will be larger for smaller droplets. Anyhow, highspeed-IR cameras are suitable to even detect the temperature gradient between fast-freezing supercooled droplets, see, e.g., refs. 2 and 4. Also, Raman spectroscopy has been applied by others to determine the temperature of (supercooled) water by analyzing the O–H stretching bands (between 3,000 and 3,600 cm^−1^) (5, 9). The same approach is expected here as well, at least until the droplet is mainly frozen (in figure 2E until 50 s).

The description and the conclusions of the freezing mechanism of supercooled droplets by Biwas et al. is unclear (“pseudoheterogeneous”) and not in agreement with other studies including refs. 2–4 and misleading, as the results are not compared. The rapidly forming dendritic ice lets the droplet suddenly (in milliseconds) appear white (or merely opaque) due to its reflectivity (and not due to a change of density) (2–4), before the remaining liquid freezes in the following seconds. According to previous work and fundamental understanding, the droplet surface temperature should be very close to 0 °C during this period. Figure 2 D and E seem to suggest a continuous cooling however.

Overall, we are not convinced by the presented temperature measurements and the proposed freezing mechanism. Nevertheless, the design of the experimental setup appears to have its strength in the combination of several analysis methods.
